# Fluorophore labelled BVDV: a novel tool for the analysis of infection dynamics

**DOI:** 10.1038/s41598-019-42540-z

**Published:** 2019-04-12

**Authors:** Christiane Riedel, Benjamin Lamp, Hann-Wei Chen, Manuela Heimann, Till Rümenapf

**Affiliations:** 1 Institute of Virology, Department of Pathobiology, University of Veterinary Medicine Vienna, Vienna, Austria; 2 Institute of Anatomy, Faculty of Veterinary Medicine, Justus-Liebig University, Giessen, Germany

## Abstract

Genetic labelling of viruses with a fluorophore allows to study their life cycle in real time, without the need for fixation or staining techniques. Within the family *Flaviviridae*, options for genetic labelling of non-structural proteins exist. Yet, no system to genetically label structural proteins has been put forward to date. Taking advantage of a previously described site within the structural protein E2, a fluorophore was introduced into a cytopathogenic (cpe) BVDV-1 virus (BVDV_E2_fluo_). This insertion was well tolerated, resulting in a 2-fold drop in titer compared to the parental virus, and remained stably integrated into the genome for more than 10 passages. The fluorophore E2 fusion protein was readily detectable in purified virus particles by Western blot and fluorescence microscopy and the particle integrity and morphology was confirmed by cryo electron microscopy. The same integration site could also be used to label the related Classical swine fever virus. Also, BVDV_E2_fluo_ particles bound to fluorophore labelled CD46 expressing cells could be resolved in fluorescence microscopy. This underlines the applicability of BVDV_E2_fluo_ as a tool to study the dynamics of the whole life cycle of BVDV in real time.

## Introduction

Bovine viral diarrhoea virus (BVDV) is an economically important pathogen within the family *Flaviviridae*, genus *Pestivirus* and has been extensively used as a model for hepatitis C virus research. Its single stranded, positive sense RNA genome encodes for one open reading frame, which is co- and post-translationally processed into 12 proteins by viral and cellular proteases. BVDV particles consist of a nucleocapsid, composed of core protein and the viral genome, and a viral envelope, in which three surface glycoproteins, E^rns^, E1 and E2, are anchored.

BVDV enters its host cells after interaction with cell surface glycosaminoglycans^1^, which is mediated by E^rns^, and binding to its cellular receptor, CD46, via E2^2,3^. After clathrin dependent endocytosis^3–5^, fusion occurs in the endosome at an acidic pH < 6^5,6^, resulting in the release of the nucleocapsid into the cytoplasm. Yet, the attachment and entry of BVDV or any other pestivirus has never been analysed in real time as suitable labelled viruses have been lacking to date.

To gain dynamic information of virus entry, viruses can be genetically labelled by the fusion of a structural protein with a fluorophore or by labelling components of intact virus particles with a fluorescent dye (e.g. adenovirus/enveloped particles with DiD) (reviewed in^7^).

Within the *Flaviviridae*, infection dynamics have been analysed for DiD labelled Dengue virus, revealing diffusion patterns on the cell surface and the recruitment dynamics of endosomal components during virus entry^8^. Although systems for genetic labelling exist within this family (^9–13^, reviewed for HCV in^14^), no option to generate genetically labelled virus particles that allow single particle visualisation does exist.

In the present paper we describe the generation and characterisation of BVDV particles that are labelled with a fluorophore fused to E2 surface glycoprotein and their applicability for the imaging of infection dynamics.

## Results

**Figure 1:**
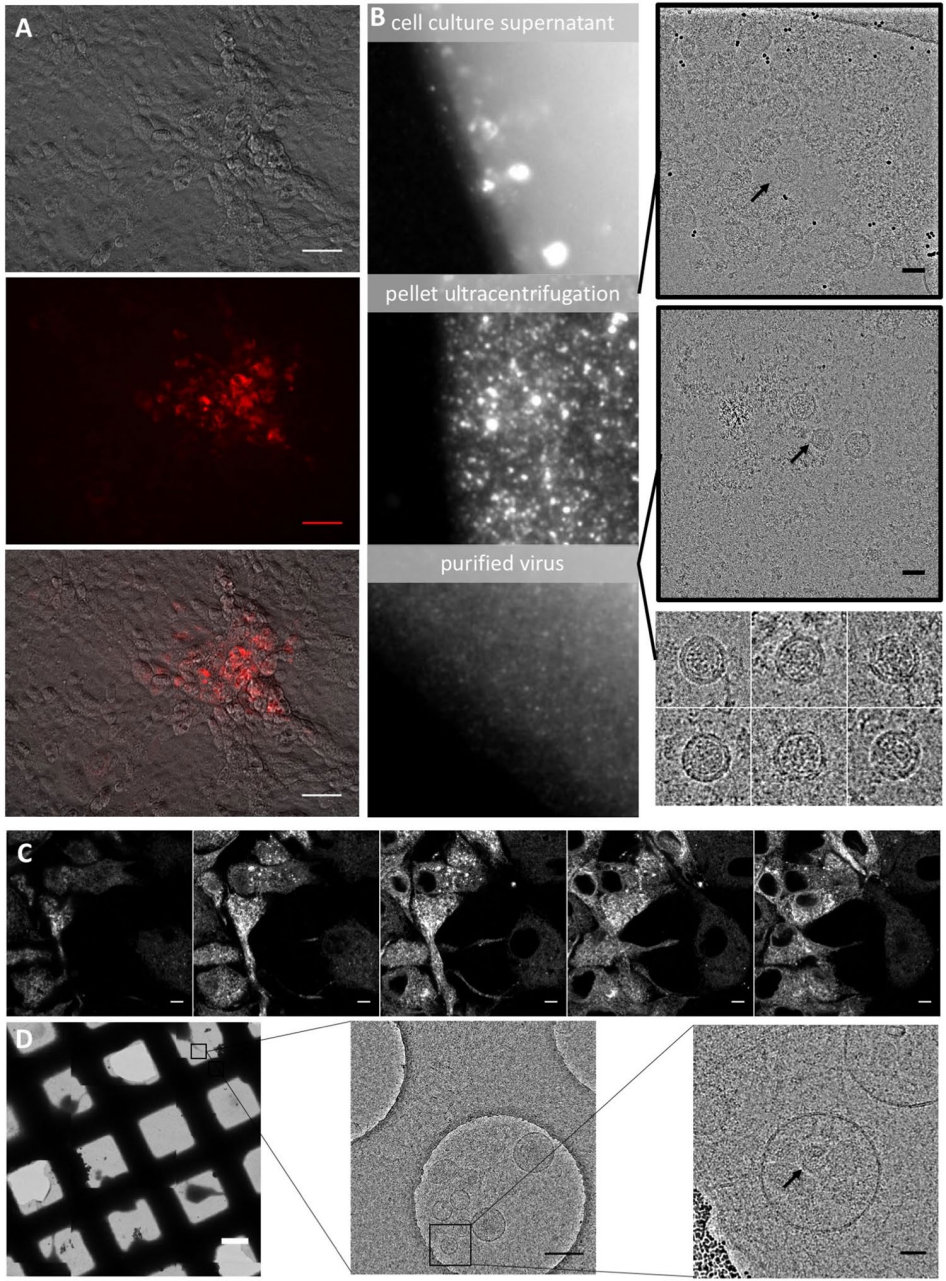
Analysis of a BVDVE2_fluo (tagged with mCherry) by fluorescence and cryo electron microscopy. (**A**) Bright field and fluorescence microscopy of BVDV_E2_fluo_ plaques 72 h post infection. Scale bar represents 50 µm. (**B**) Fluorescence microscopy (left column) and cryo electron microscopy (right column) of BVDV_E2_fluo_ during and after purification from cell culture supernatant. Arrows indicate virus particles; scale bar represents 50 nm. Box size of electron micrographs of individual virus particles is 100 nm. (**C**) Confocal Z-stack of fluorescent micrographs through BVDV_E2_fluo_ infected MDBK cells 48 h after infection. Scale bar represents 5 µm. (**D**) Cryo electron micrographs of BVDV_E2_fluo_ infected cells 48 h after infection. Arrow indicates an intracellular virus particle. Scale bars from left to right represent 50 µm, 500 nm and 50 nm.

Positions within BVDV E2 and E^rns^ that tolerate insertions of a flag-tag were previously described by Wegelt *et al*.^15^. We sought to exploit this property by insertion of a fluorescent protein sequence (mCherry or mClover) after the first amino acid (aa) residue of the E2 coding sequence of a cytopathogenic BVDV1 (strain C87, BVDV_E2_fluo_). Rescue of *in vitro* transcribed genome of BVDV_E2_fluo_ was attempted by electroporation in MDBK cells. 24 h after transfection, a specific fluorescence signal could be detected in the cytoplasm and a cytopathogenic effect (cpe) was visible starting from 48 h after transfection (data not shown). Infectious progeny virus was released in the supernatant, with an average titer of 6.8 × 10^5^ ffu/ml 48 h after transfection (n = 4). The average titer of the parental virus after 48 h was 1.3 × 10^6^ ffu/ml. The presence of the fluorophore – E2 fusion protein could be demonstrated in cell lysate and concentrated virus particles by Western blot analysis detecting either mClover or E2 (Sup. Fig. 1). Fluorescence positive foci could be detected after infection of MDBK cells (Fig. 1A). BVDV_E2_fluo_ could be propagated for more than 10 passages without evidence of loss of fluorophore expression. In analogy to BVDV, a Classical swine fever virus (CSFV) with a GFP label at the N-terminus of E2 (CSFV__E2_GFP_) was generated employing the reverse genetic clone of the CSFV strain Alfort Tübingen (GenBank: J04358.2). Also, in the context of CSFV, fluorophore expression could readily be observed in transfected cells and a size shift of E2 was detectable by Western blot analysis in the lysate of infected cells and concentrated virus particles (Sup. Fig. 2). CSFV__E2_GFP_ showed no loss of GFP expression over 10 passages but titres were reduced 12-fold in comparison to the parental virus.

To examine whether fluorophore labelled E2 incorporated into virus particles would result in the emission of a specific fluorescence signal, virus containing supernatant was analysed by fluorescence microscopy before, during and after purification by ultracentrifugation and molecular filters. In the unconcentrated supernatant and the resuspended pellet after ultracentrifugation, strong, inhomogeneous signals were observed (Fig. 1B, upper and middle panel), which most likely correspond primarily to cellular debris. Analysis of the pellet after ultracentrifugation by cryo electron microscopy primarily showed inhomogeneous proteinaceous aggregates and vesicles but also membrane surrounded, nucleocapsid containing particles of about 50 nm diameter (Fig. 1B). The morphology of observed virus particles closely resembled already published cryo electron micrographs of BVDV^16^. After further purification, fine homogeneous fluorescent foci were observed (Fig. B, lower panel), which likely represent single virus particles. Employing cryo electron microscopy, virus particles could readily be identified at this purification step.

Closer analysis of the distribution pattern of fluorophore labelled E2 within MDBK cells infected for 48 h demonstrated the emission of fluorescence not only from perinuclear regions of the cytoplasm, but also from cellular protrusions that frequently were in contact with neighbouring cells (Fig. 1C). To assess the possibility that this signal might partially be emitted from fully assembled particles, we analysed these cellular protrusions by cryo electron microscopy of MDBK cells infected for 48 h with a MOI of 1. Virus-like particles, corresponding morphologically to particles observed in purified cell culture supernatant, could indeed be identified inside vesicles. This is in accordance with the presence of pestiviral particles inside vesicles of the secretory pathway as observed by Schmeiser *et al*.^17^. Hence, BVDV_E2_fluo_ can be employed to track and examine virus egress.

**Figure 2:**
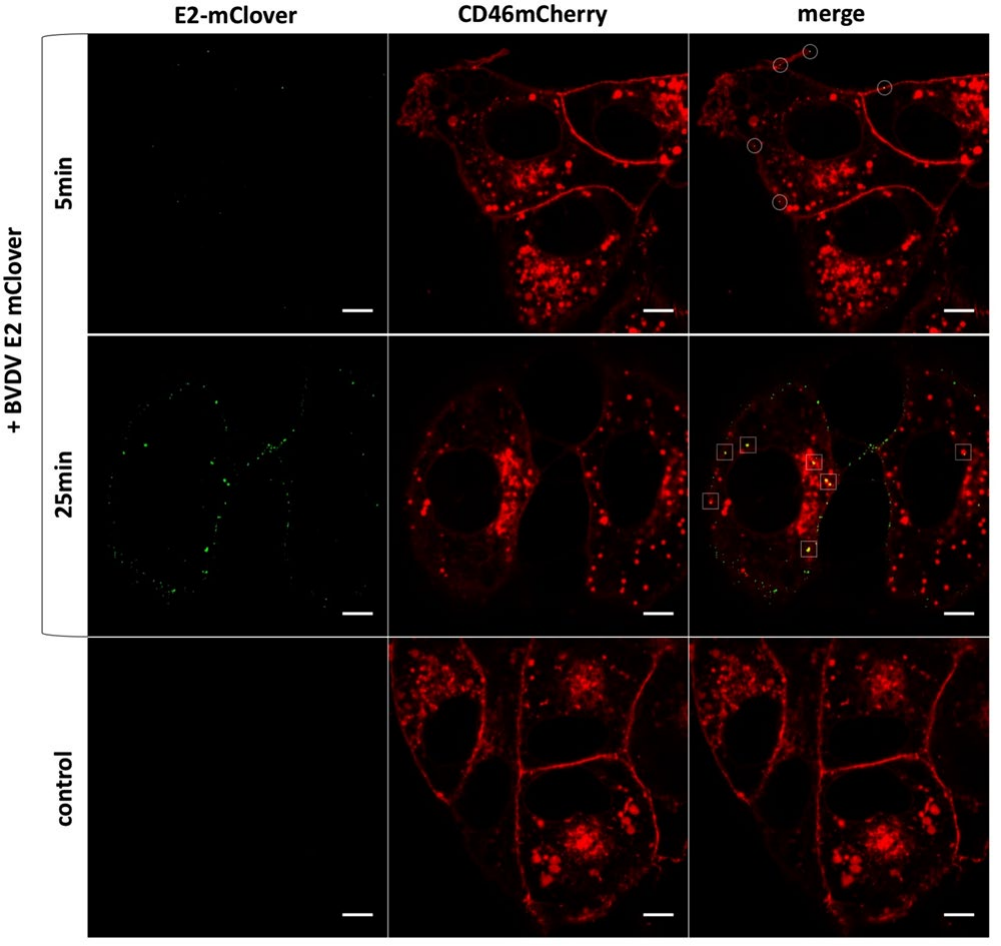
BVDV_E2_fluo_ can be visualized by confocal fluorescence microscopy interacting with SK6_CD46_fluo cells. A representative Z-slice through SK6_CD46_fluo cells 5 or 25 min after addition of BVDV_E2_fluo_ at a MOI of 1000 is shown. Fluorescent foci in contact with the CD46_fluo positive cellular surface 5 min after virus addition are indicated by white circles. Fluorescent foci colocalizing with intracellular, CD46_fluo positive vesicles 25 min after virus addition are indicated by white squares. Scale bar is 5 µm. Control = no addition of virus.

To examine the applicability of BVDV_E2_fluo_ for analysis of virus entry, SK6 cell lines inducibly expressing fluorophore labelled bovine CD46 (SK6_CD46_fluo, mCherry or mClover) were generated. After induction of expression, the fluorescence signal emitted by SK6_CD46_fluo was primarily localised in the cell membrane and intracellular vesicles. The fluorescent protein was inserted after the 4^th^, C-terminal complement control protein (CCP) module at a position previously described for human CD46^18^. As the binding site of BVDV E2 is located in the N-terminal CCP1 module^3^ and the distance between the end of CCP1 (E97) and the end of CCP4 (K285) is 85 Å in the crystal structure of human CD46 (PDB:3o8e), an inhibitory effect of the fluorophore on virus binding is unlikely. Expression of CD46_fluo within SK6 cells indeed increased their permissiveness for BVDV_E2_fluo_ and the parental virus by more than 50-fold, which is in accordance with previous data^3^ and indicates that CD46_fluo retains its function as an entry receptor. SK6_CD46_fluo were incubated with a MOI of 1000 and fixed at defined time points after virus addition. Distinct fluorescent foci appeared on the cell surface starting 5 min after virus addition (Fig. 2, upper panel) and their number increased until 25 min after infection (Fig. 2, middle panel). At 25 min after infection, fluorescent spots could also be identified in CD46 positive vesicles inside the cell. These findings demonstrate the applicability of BVDV_E2_fluo_ as a tool to study the real time dynamics of pestivirus entry.

## Discussion

Our knowledge of viral lifecycles is often derived from experiments that require the disruption/fixation of biological entities or use an indirect/temporarily separated read-out system. The results of these experiments have allowed us to dissect pathways and molecules important for different stages of the viral life cycle. Yet, they are not well suited to examine process dynamics.

With the generation of BVDV_E2_fluo_, we now possess a tool to follow the pestiviral life cycle in real time. The growth performance of BVDV_E2_fluo_ is comparable to the parental virus and ultrastructural analysis did not reveal obvious morphological changes of the virus particles. This suggests that the behaviour of BVDV_E2_fluo_ is also representative of unlabelled BVDVs. The finding that CSFV can be modified in the same way suggests that the chosen integration site might be tolerated by other pestiviral species. When analysed in the context of the E2 crystal structure, the location of the fluorophore at the very N-terminus of the protein likely provides enough distance to the β-hairpin located in domain 2, which has been proposed to be involved in receptor binding^19,20^. Yet, we can only speculate whether the slightly reduced growth rate of BVDV_E2_fluo_ is the result of sterical hindrance during receptor interaction or can be attributed to the increase in genome size. BVDV_E2_fluo_ will be a valuable tool to further examine virus entry by evaluating preferred sites of viral attachment, mean diffusion/transport path and time on the cell surface, time from endosomal uptake to fusion and site and pH of fusion. Furthermore, mechanisms of virus egress can now be dissected in real time employing inhibitors. Correlation of fluorescence and cryo electron microscopy, potentially in combination with cryo focus ion beam milling, will allow the ultrastructural examination of subcellular compartments that contain E2_fluo.

Also, BVDV_E2_fluo_ could proof valuable to simplify serodiagnostic procedures such as serum neutralisation tests.

## Materials and Methods

### Cell culture

Cells used in this study were propagated in DMEM (Gibco, Waltham, USA) containing 10% chromatographed FCS (Bio&Sell, Feucht, Germany) and penicillin/streptomycin at 37 °C. For SK6 CD46_fluo cells, 100 µg/ml G418 (Gibco) and 1 µg/ml puromycin (Sigma-Aldrich, St.Louis, USA) were added to the medium.

### Construction of BVDV_E2_fluo_ and CD46_fluo

For the insertion of a fluorophore after the first amino acid of E2 (H693), a BssHII and a SbfI site were introduced by PCR (Phusion, NEB, Ipswich, USA) (forward primer: aaacctgcaggCTAGACTGCAAACCTGAATAC; reverse primer: tttgcgcgcGTGCCCTTGCACCCCTGTTATCAG). Fluorophores were introduced into the full-length clone of the cpe BVDV1 strain C87 using these restriction sites after PCR amplification (forward primer: aaagcgcgcATGGTGAGCAAGGGCGAG; reverse primer: tttcctgcaggCTTGTACAGCTCGTCCATG). The SbfI site was subsequently removed by PCR to restore the open reading frame (forward primer: ggaCTAGACTGCAAACCTGAATAC; reverse primer: gctaccCTTGTACAGCTCGTCCATG). The mCherry sequence was derived from the plasmid pmCherry (Clontech, Mountain View, USA) in which the SbfI site present in the mCherry coding region was destroyed by PCR (forward primer: CAGGACTCCTCttTGCAGGAC; reverse primer: GTCCTGCAaaGAGGAGTCCTG). MClover3^21^ (GenBank: KX987298.1) was ordered as a synthetic gene (IDT, Coralville, USA). In the context of CSFV, a NcoI site and a MluI site were introduced by PCR after the first amino acid of E2 (forward primer: aaaccatggCTAGCCTGTAAGG, reverse primer: tttacgcgtCCGCCCTTGTGC) and eGFP (peGFP-N1, Clontech) was cloned into these sites after PCR amplification.

To label bovine CD46, a NdeI and MluI site were introduced after aa 294 at the homologous position previously described by Crimeen-Irwin *et al*.^18^ for human CD46 employing the following primer pair: forward: aaaacgcgtCCTAACGGTGCTGAGGGTTTAG; reverse: tttcatatgTTTAATACACTTTGGTAGCTC. Fluorophores were inserted into these sites by standard cloning procedures after PCR amplification (forward primer: aaacatATGGTGAGCAAGGGCGAGGAG; reverse primer: tttacgcgtCTTGTACAGCTCGTCCATG).

### Generation of CD46_fluo expressing cell lines

SK6 tet-on cells were transfected with linearized plasmid encoding CD46_fluo after the Tet-on responsive element using electroporation. Clonal populations of fluorescence positive cells were selected employing 2 µg/ml puromycin and selected clones propagated for use in further experiments.

### Virus production and purification

Initial virus stocks were generated by electroporation of *in vitro* transcribed BVDV_E2_fluo_ genomes into MDBK cells. Briefly, cDNA was transcribed with SP6-RNA-polymerase (NEB, Ipswich, USA) and 2.5 µg of RNA were transfected in 5 × 10^6^ cells with a BioRad GenePulser. Viruses were then further propagated by passaging on MDBK cells every 3–4 days. For the generation of highly concentrated virus stocks, one five-layer tissue culture flask (Corning, Corning, USA) was seeded with 1 × 10^8^ MDBK cells and infected at a MOI of 0.1. Supernatant was harvested first after 48 h, and subsequently every 24 h until a complete cpe was observed. Cellular debris was removed from the supernatant by centrifugation at 4000xg for 10 min. Afterwards, virus particles contained in 300 ml supernatant were pelleted through a 15% Sucrose cushion for 3 h at 35,000 rpm in a Beckmann 45Ti rotor. Pellets were resuspended overnight in DMEM, and small contaminants were removed by a HisTrap CaptoCore 700 column (GE Life Sciences, Marlborough, USA). Subsequently, the virus was additionally concentrated by a 100 kDa centrifugal concentrator (Sartorius, Göttingen, Germany) to a final volume of 50–100 µl.

### Western blot analysis

Western blot analysis was essentially performed as described in^22^. BVDV E2 was detected employing the mouse monoclonal antibody 6A5, CSFV E2 employing the mouse monoclonal antibody A18 and mClover employing the antibodies 7.1 and 13.1 against GFP (Roche, Basel, Switzerland). The chemiluminescence signal was detected by a Licor c-digit.

### Fluorescence microscopy

5 × 10^3^ MDBK or SK6 CD46_fluo cells were seeded in each well of a IBIDI µ slide 2 well coculture slide (IBIDI, Martinsried, Germany) in 50 µl medium. For the analysis of signal distribution in infected cells, MDBK cells were infected with BVDV_E2_fluo_ with a MOI of 1 and fixed using 4% paraformaldehyde in PBS 48 h after infection. For the analysis of binding of BVDV_E2_fluo_ to SK6 CD46_fluo cells, approximately 1000 infectious particles of BVDV_E2_fluo_ were added per cell and the experiment was stopped by fixation 2 min, 5 min, 10 min, 15 min, 20 min and 25 min after virus addition. CD46_fluo expression was induced 16 h before infection employing 500 ng/ml doxycyclin. Data were acquired as Z-stacks on a Zeiss AiryScan microscope at 63x magnification with an oil immersion objective and images processed with the Zen software package. ImageJ was used for data analysis^23^.

Virus preparations were imaged on an Olympus IX70 equipped with an Olympus X10 camera at 100x magnification with an oil immersion objective.

### Cryo electron microscopy

2/1 200mesh copper grids (Quantifoil, Großlöbichau, Germany) were glow discharged and 5 µl of concentrated BVDV_E2_fluo_ were added. Excess liquid was removed by manual blotting and the sample vitrified in a 1/3 ethane 2/3 propane bath cooled to <180 °C. For the analysis of infected cells, 5 × 10^3^ MDBK cells were seeded on Quantifoil 2/1 200 mesh gold grids previously coated with poly-L-lysine (Sigma). 24 h after seeding, cells were infected with a MOI of 1 with BVDV_E2_fluo_. 48 h after infection, grids were checked by fluorescence microscopy to confirm emission of specific fluorescence signal by all cells on the grid. Subsequently, samples were vitrified as described above. Samples were analysed on a FEI Tecnai F30 Polara equipped with a K2 Summit direct electron detector (Gatan) at 300 kV with a dose of 10–20 e^-^/Å^2^ and a pixel size of 1.3 to 1.7 Å. Acquired movies were aligned within SerialEM and images further processed employing the IMOD^24^ and bsoft^25^ software suits.

## Supplementary information

Supplementary Dataset 1
