# Supplementary material for: Fluorophore labelled BVDV: a novel tool for the analysis of infection dynamics
Source: Sci Rep. 2019 Apr 12;9:5972. doi: 10.1038/s41598-019-42540-z (PMC6461705; doi:10.1038/s41598-019-42540-z)

Fluorophore labelled BVDV: a novel tool for the analysis of infection dynamics

Christiane Riedel[1]*, Benjamin Lamp[1], Hann-Wei Chen[1], Manuela Heimann[2], Till Rümenapf[1]

[1]Institute of Virology, Department of Pathobiology, University of Veterinary Medicine Vienna, Vienna, Austria.

[2]Institute of Anatomy, Faculty of Veterinary Medicine, Justus-Liebig University, Giessen, Germany.

*corresponding author: christiane.riedel@vetmeduni.ac.at

Supplementary Figure 1

Detection of E2 and E2_fluo in virus infected cells and concentrated virions. Western Blot analysis of BVDVE2_fluo (labelled either with mCherry or mClover) infected cells and concentrated particles detecting either E2 protein or GFP. Cells infected with the parental virus and concentrated particles of said virus serve as control. The bands corresponding to E2 mono- and dimers are indicated. E2 = E2 monomer; E1-E2 = E1-E2 heterodimer; E2-E2 = E2 homodimer; E2_fluo = fluorophore labelled E2; αE2 = detection of E2; αGFP = detection of GFP. The reason for the slightly smaller molecular weight of the mCherry E2 monomer is unknown.

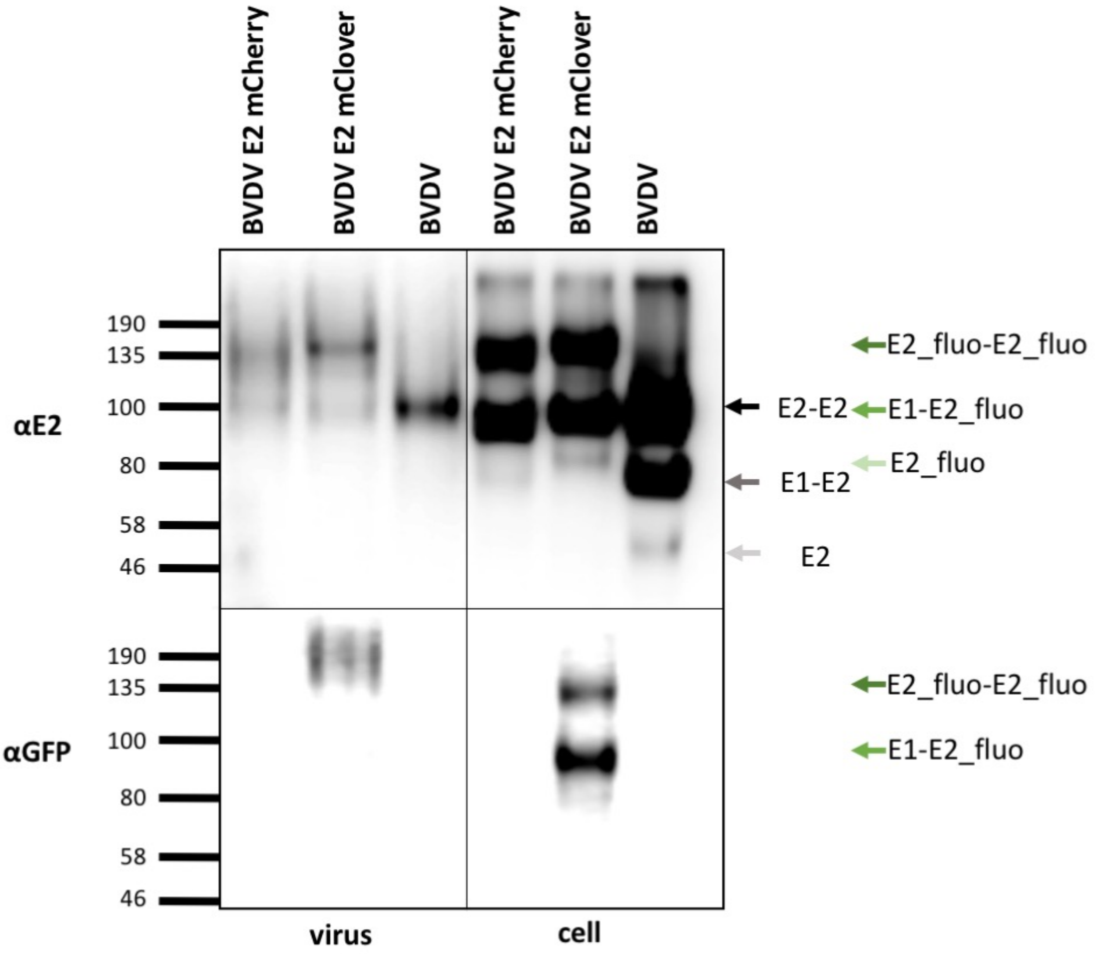

Supplementary figure 2:

Characterisation of a CSFV with a fluorophore label at the E2 N-terminus. (A) Brightfield and fluorescence microscopy of CSFV$_{_E2_GFP}$ 24h after electroporation of in vitro transcribed genomic RNA into SK6 cells. (B) Western blot analysis of lysates of SK6 cells infected with either CSFV or CSFV$_{_E2_GFP}$ and concentrated virions of said viruses. The viral E2 protein was detected employing the monoclonal antibody A18. p1 = virus in passage 1; p10 = virus in passage 10. Bands including unlabelled E2 are indicated by black asterisks and bands including labelled E2 are indicated by green asterisks. * = E2 monomer, ** = E1-E2 heterodimer, *** = E2-E2 homodimer (C) Growth curve of the parental CSFV and CSFV$_{_E2_GFP}$ after infection of SK6 cells with an MOI of 0.01. Depicted are the mean viral titres of 3 independent experiments.

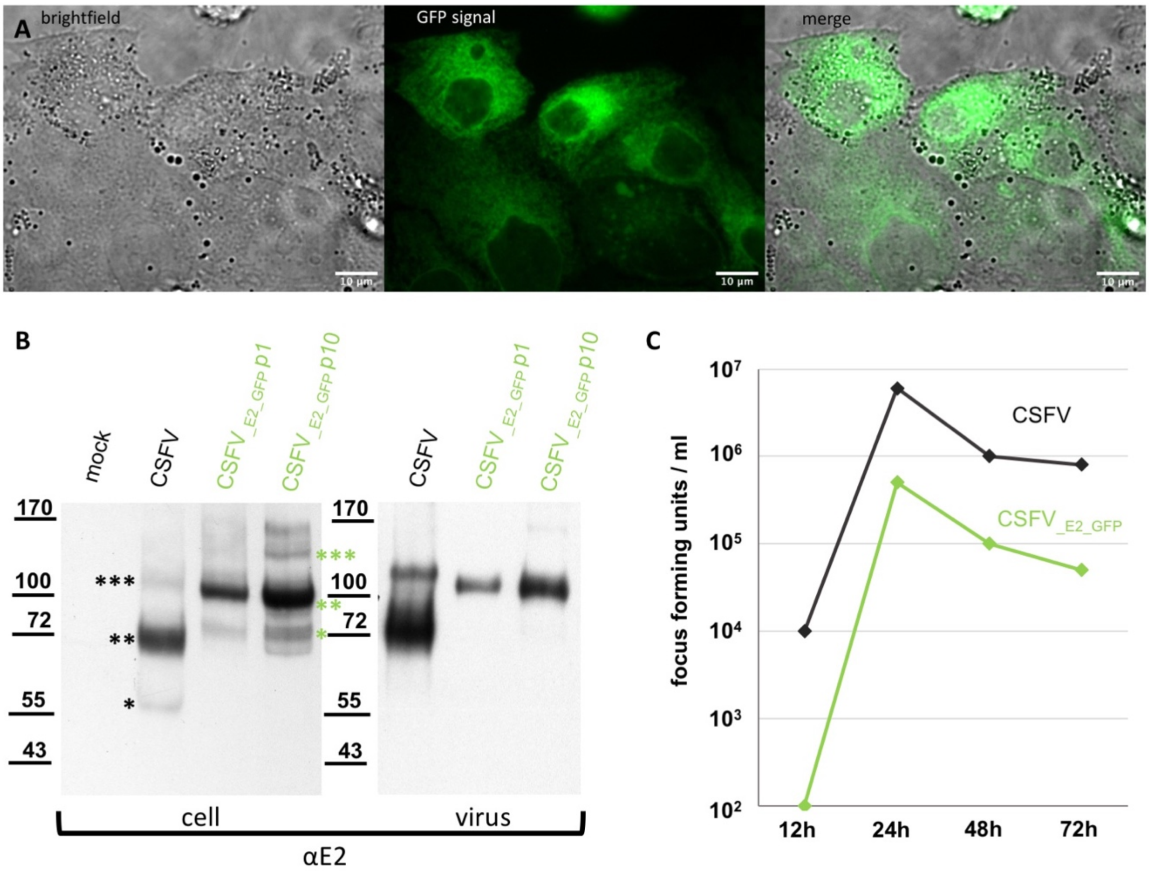

Supplement: Supplementary file 1 — Supplementary Dataset 1 [file 41598_2019_42540_MOESM1_ESM.pdf]
